# Effectiveness of physiotherapy exercise following total knee replacement: systematic review and meta-analysis

**DOI:** 10.1186/s12891-015-0469-6

**Published:** 2015-02-07

**Authors:** Neil Artz, Karen T Elvers, Catherine Minns Lowe, Cath Sackley, Paul Jepson, Andrew D Beswick

**Affiliations:** Peninsula Allied Health Centre, School of Health Professions, University of Plymouth, Plymouth, PL6 8BH UK; Musculoskeletal Research Unit, School of Clinical Sciences, University of Bristol, Bristol, BS10 5NB UK; Physiotherapy Research Unit, Oxford United Hospitals NHS Trust, Oxford, UK; King’s College London, Capital House, Guy’s Campus, London, SE1 3QD UK; School of Sport, Exercise and Rehabilitation Sciences, University of Birmingham, Edgbaston, Birmingham, B15 2TT UK; Musculoskeletal Research Unit, School of Clinical Sciences, University of Bristol, Bristol, BS10 5NB UK

**Keywords:** Systematic review, Meta-analysis, Rehabilitation, Physiotherapy, Total knee replacement, Arthroplasty, Exercise, Osteoarthritis, Outcome

## Abstract

**Background:**

Rehabilitation, with an emphasis on physiotherapy and exercise, is widely promoted after total knee replacement. However, provision of services varies in content and duration. The aim of this study is to update the review of Minns Lowe and colleagues 2007 using systematic review and meta-analysis to evaluate the effectiveness of post-discharge physiotherapy exercise in patients with primary total knee replacement.

**Methods:**

We searched MEDLINE, Embase, PsycInfo, CINAHL and Cochrane CENTRAL to October 4^th^ 2013 for randomised evaluations of physiotherapy exercise in adults with recent primary knee replacement.

Outcomes were: patient-reported pain and function, knee range of motion, and functional performance. Authors were contacted for missing data and outcomes. Risk of bias and heterogeneity were assessed. Data was combined using random effects meta-analysis and reported as standardised mean differences (SMD) or mean differences (MD).

**Results:**

Searches identified 18 randomised trials including 1,739 patients with total knee replacement. Interventions compared: physiotherapy exercise and no provision; home and outpatient provision; pool and gym-based provision; walking skills and more general physiotherapy; and general physiotherapy exercise with and without additional balance exercises or ergometer cycling.

Compared with controls receiving minimal physiotherapy, patients receiving physiotherapy exercise had improved physical function at 3–4 months, SMD −0.37 (95% CI −0.62, −0.12), and pain, SMD −0.45 (95% CI −0.85, −0.06). Benefit up to 6 months was apparent when considering only higher quality studies.

There were no differences for outpatient physiotherapy exercise compared with home-based provision in physical function or pain outcomes. There was a short-term benefit favouring home-based physiotherapy exercise for range of motion flexion.

There were no differences in outcomes when the comparator was hydrotherapy, or when additional balancing or cycling components were included. In one study, a walking skills intervention was associated with a long-term improvement in walking performance. However, for all these evaluations studies were under-powered individually and in combination.

**Conclusion:**

After recent primary total knee replacement, interventions including physiotherapy and exercise show short-term improvements in physical function. However this conclusion is based on meta-analysis of a few small studies and no long-term benefits of physiotherapy exercise interventions were identified. Future research should target improvements to long-term function, pain and performance outcomes in appropriately powered trials.

**Electronic supplementary material:**

The online version of this article (doi:10.1186/s12891-015-0469-6) contains supplementary material, which is available to authorized users.

## Background

In the year to 31st March 2013, over 75,000 primary total knee replacements were performed by the NHS in England and Wales with about 97% of procedures subsequent to osteoarthritis [1]. In the USA in 2010, the estimated number of hospital discharges after total knee replacement procedures was 719,000 [2]. Osteoarthritis is the leading cause of pain and disability in older people [3,4] and if pharmacological and conservative treatments do not relieve symptoms joint replacement is recommended [5].

Rehabilitation, with a particular emphasis on physiotherapy and exercise, is widely promoted after total knee replacement [6]. During the hospital stay, physiotherapy targets mobilisation and achievement of functional goals relating to hospital discharge. Further post-discharge physiotherapy and exercise-based interventions promote re-training and functional improvement. However, provision of these services varies in content and duration [7,8].

Minns Lowe and colleagues reviewed evidence from 6 randomised trials with 614 patients on the effectiveness of post-discharge physiotherapy after total knee replacement [9]. Since their literature search in 2007, additional trials have been published. Our aim was to update the review and further explore the possible benefit of specific physiotherapy modalities.

## Methods

We used systematic review methods as described in the Cochrane handbook of systematic reviews [10], and reported the review in accordance with the PRISMA statement for reporting systematic reviews and meta-analyses of randomised controlled trials [11].

### Types of studies

To eliminate selection bias, we included studies that were randomised controlled trials (RCTs) with randomisation either at the individual or cluster level. We also included studies with a quasi-randomised design (for example alternate allocation). Studies reported only as abstracts, or that we were unable to acquire as full text copies using interlibrary loans or email contact with authors, were excluded from the analyses. Studies where patients with total knee replacement were identified retrospectively were also excluded. No language restrictions were applied.

### Participants

Adults with recent primary total knee replacement.

### Types of interventions

We included any physiotherapy or exercise-based intervention. Interventions commenced at a pre-specified time after discharge from the hospital; typically at 2–12 weeks, and were either outpatient, community or home-based. We included studies comparing physiotherapy exercise interventions with: usual or standard care; different types of intervention including home-based; and enhanced physiotherapy formats with additional components. Interventions including electrical stimulation, acupuncture or electrical modalities such as continuous passive motion were excluded as these were considered as adjunct to physiotherapy or exercise-based intervention.

### Search methods for identification of studies

MEDLINE, Embase and PsycINFO on the OvidSP platform, CINAHL and Cochrane Library databases were searched from inception to 4th October 2013. Search terms related to: hip and knee replacement; randomised controlled trial; and exercise, rehabilitation and physiotherapy. Previous systematic reviews and meta-analyses were checked [9,12]. Citations of key articles in ISI Web of Science were checked and reference lists searched. Articles identified were managed in an Endnote X5 database.

### Inclusion/exclusion

Full articles relating to potentially relevant abstracts identified during initial screening were obtained and assessed independently for eligibility, based on the defined inclusion criteria, by two reviewers (NA, KTE). If there was any doubt a third reviewer was consulted (ADB).

### Data extraction

Data extraction was undertaken in duplicate (NA, KTE, ADB). Reasons for exclusion at this stage were summarised. Results were recorded on a piloted data extraction form and Excel spreadsheet. Data was extracted on: country and dates of study; participants (indication, age, sex); inclusion and exclusion criteria; content of intervention and comparison (control) group; setting, timing, duration and intensity of intervention; follow up duration; losses to follow up and reasons; and outcomes.

For outcomes reported as continuous variables, means and standard deviations were extracted. If outcomes were reported as means and confidence intervals, or medians and inter-quartile ranges, appropriate conversions were applied [10].

The primary author of the study was contacted for missing data if necessary. We also asked if any outcomes not reported in their publications had been collected. If authors had provided information to other reviewers this data was included in our analyses and acknowledged appropriately.

### Assessment of risk of bias in included studies

Potential sources of bias were assessed according to the Cochrane risk of bias table [10]. Bias was assessed on the grounds of: random sequence generation, allocation concealment, blinding of outcome assessment, incomplete outcome data, selective reporting, and other sources. In the context of post-surgical physiotherapy exercise, participants and therapists were generally unable to be blinded to the intervention. Quality was judged as: Good; Reasonable (e.g. non-blind follow up with self-complete questionnaires); or Possible bias (unequal or major loss to follow up, or important baseline differences).

### Data synthesis

If sufficient studies reported common outcomes, data was combined as standardised mean differences using random effects meta-analysis [10,13]. Where outcomes used the same measurement scale we combined data as the mean difference.

Heterogeneity between included studies was assessed using the I^2^ statistic. Possible heterogeneity arising from inclusion of studies of different methodological quality was investigated based on the risk of bias assessment. Funnel plots were used to explore publication bias.

## Results

### Included studies

**Figure 1:**
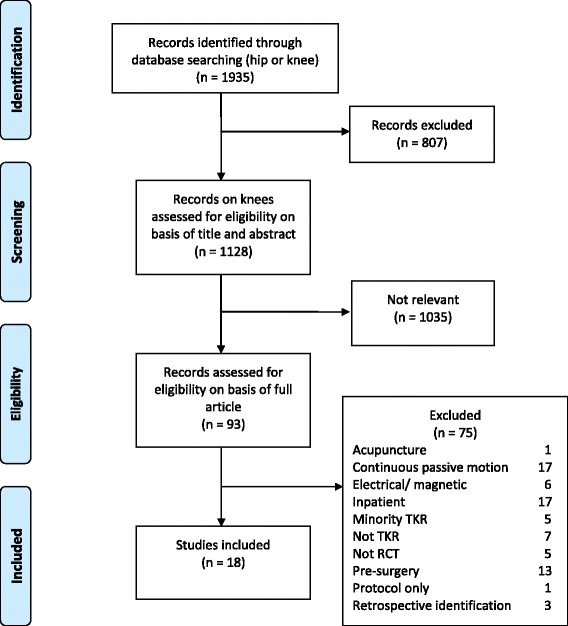
**Systematic review flow diagram.**

Review progress is summarised as a flow diagram in Figure 1. Eighteen eligible randomised controlled trials were identified. Reasons for exclusion are summarised in Additional file 1 and excluded studies are listed in Additional file 2.

Characteristics of the 18 included studies are presented in Table 1. Studies ranged in size from 43–160 patients (median 94) and included a total of 1,739 patients. Where reported, the main diagnosis was osteoarthritis, and the mean age in studies ranged from 66 to 73.5 years. The duration of follow up ranged from 3 weeks to 24 months, though we describe data in our meta-analysis from 3 months onwards.

**Table 1:** **Characteristics of included studies**

**Publication**	**Operation**	**Primary focus of intervention**	**Follow up interval**
**Location**	**Indication**	**Study setting**	**Outcomes**
**Date of study**	**Number randomised (intervention:control)**	**Intervention, health professional. Time commenced**	**Adherence to intervention**
	**Mean age (% female)**	**Timing, duration and intensity**	**Losses to follow up (intervention: control)**
		**Control group care**	
Bruun-Olsen et al. 2013 [29] Norway 2008-2010	Primary TKA	Walking skills	On completion of intervention and 9 months after intervention
	Osteoarthritis	Outpatient physiotherapy department	KOOS, 6 minute walk test, performance tests, ROM, self-efficacy in activities
	N = 57 (29:28)	Physiotherapist-led walking-skills programme with emphasis on weight-bearing exercises. Commenced 6 weeks after surgery	28/29 completed programme (97%) 6 (2:4) not followed up
	69 (56.1%)	6–8 weeks	
		Usual physiotherapy	
Evgeniadis et al. 2008 [19] Greece 2006	Primary TKA	Strengthening	6, 10 and 14 weeks after surgery
	Osteoarthritis	Home	SF-36, Iowa Level of Assistance Scale, active ROM
	N = 48 (24:24)	Supervised exercise programme with emphasis on strengthening lower extremities	20/24 completed programme (83%)
	69 (56.3%)	Commenced after hospital discharge	13 (9:4) not followed up
		8 weeks	
		Control received standard preoperative and postoperative care	
Frost et al. 2002 [17] UK 1995-1996	Primary unilateral TKA	Functional exercise	3, 6 and 12 months
	Osteoarthritis	Home	VAS pain, ROM, leg extensor power, walking speed, gait speed
	N = 47 (23:24)	Warm up exercise, chair rise, walking, and leg lifts. Commenced after hospital discharge	16/23 completed programme (70%)
	71.3 (48.9%)	Number of visits and duration not specified	20 (7:13) not followed up
		Controls given instructions to continue exercises taught in hospital	
Fung et al. 2012 [27] Canada 2009-2010	TKA	Balance and posture control additional to outpatient physiotherapy	Discharge from physiotherapy, estimate about 3 months
	Not specified	Outpatient department in rehabilitation hospital	ROM, 2-minute walk test, NRS pain, LEFS, Activity-specific Balance Confidence Scale, length of rehabilitation, satisfaction
	N = 50 (27:23)	Wii Fit gaming activities focused on multidirectional balance, and static and dynamic postural control	27/27 completed programme (100%)
	68.1 (66%)	Commenced a mean of 38–47 days after surgery	0 lost to follow up
		Twice weekly for mean of about 8 weeks	
		All patients received twice-weekly outpatient physiotherapy. Control patients also received 15 minutes of lower extremity strengthening and balance training exercises	
Harmer et al. 2009 [30] Australia 2005-2006	Primary TKA	Hydrotherapy compared with gym-based therapy	8 and 26 weeks
	Not specified	Community pool	WOMAC, VAS, 6 minute walk test, stair ascent, ROM, knee oedema
	N = 102 (53:49)	Supervised classes in pool with walking forward and backward, stepping sideways, step-ups, jogging, jumping, kicking, knee ROM exercises, lunges, and combined squats and upper extremity exercises.	81% of patients attended at least 8/12 sessions 3 (2:1) lost to 26 week follow up
	68.3 (57%)	Commenced 2 weeks after surgery	
		Twice a week, 60 min duration for 6 weeks	
		Control patients received gym-based rehabilitation with ergometer cycling; walking on a treadmill; stair climbing; standing isometric, balance and knee ROM exercises at a bar; and sit to stand exercises	
Kauppila et al. 2010 [13] Finland 2002-2005	Primary unilateral TKA	Multidisciplinary rehabilitation programme	2 months, 6 months, 12 months
	Osteoarthritis	University hospital outpatient department	WOMAC, 15 min walk test, stair ascent/ descent test, isometric strength, ROM
	N = 86 (44:42)	Week 1: physiotherapist assessment; 3 group sessions (45 minutes) with lower limb strengthening exercises; 2 pool gymnastic sessions (30 minutes) with lower limb stretching and mobility, and functional exercises focused on walking; lectures by social worker (60 minutes) and nutritionist (90 minutes)	44/44 attended multidisciplinary rehabilitation programme (100%)
	70.6 (75.6%)	Week 2: 2 lower limb strengthening exercise group sessions (45 minutes); 3 pool gymnastic sessions (45 minutes); orthopaedic surgeon lecture (45 minutes) and clinical assessment (15 minutes).	11 (8:3) lost to 6 and 12 month follow up
	Included 60–80 years	Daily supervised group stretching exercises (30 minutes)	
		Twice weekly supervised group Nordic walking (30 minutes)	
		4 group rehearsals of relaxation strategies (30 minutes)	
		Individualised exercise recommendations (40 minutes).	
		2 group sessions on coping strategies (90 minutes) and individual visit with psychologist	
		Total 10 days at 2–4 months after surgery	
		Control received an exercise programme to complete at home from 2 months after surgery.	
Kramer et al. 2003 [25] Canada Not specified	Primary unilateral TKA	Basic and advanced ROM and strengthening exercises.	12, 26 and 52 weeks
	Osteoarthritis	Home- and clinic- based groups	WOMAC, SF-36, KSS, stair ascent and descent, 6 minute walk test
	N = 160 (80:80)	Attended outpatient physical therapy. Therapists able to modify or add exercises, use therapeutic modalities, joint mobilisations or other measures as appropriate	154/160 complete programmes (96%)
	68.4 (56.9%)	Between 2 to 12 weeks after surgery, two sessions per week for 1 hour per session	26 (11:15) medical issues, withdrawn consent
		Home-base group received a telephone call once in week 2 to 6 and once in weeks 7–12 reminding them of the importance of exercise and to give advice	
Liebs et al. 2010 [28] Germany 2005-2006	Primary unilateral TKA	Ergometer cycling (additional to standard programme)	3, 6, 12 and 24 months
	Osteoarthritis or osteonecrosis	Multiple hospitals	WOMAC, SF-36 PCS, patient satisfaction
	N = 159 (85:74)	Cycling with minimal resistance under guidance of a physical therapist. Aim was to improve muscle coordination, proprioception and ROM.	No information on patient adherence reported
	69.8 (71.7%)	Three times a week for at least three weeks, starting after the second postoperative week	24 (10:14) lost to follow up at 3 months
		Controls received standard physiotherapy programme only	
Madsen et al. 2013 [24] Denmark 2010-2011	Fast-track primary TKA	Group-based programme compared with home-based programme	3 and 6 months
	Osteoarthritis	Physiotherapist led strength endurance training, education, patient discussion. Home exercises twice weekly with strength training, endurance training on exercise bike, walking, balance, training and muscle strength training.	OKS, SF-36 physical function, EQ-5D, ROM, peak Leg Extensor Power, balance test, 10 m walk test, sit-to-stand tests, VAS pain during Leg Extensor Power test.
	N = 80 (40:40)	2 sessions per week for 6 weeks starting 4–8 weeks after surgery. Average 10.5 sessions (range 4–12)	Patients in group-based programme attended mean 10.5 sessions (range 4–12). Adherence to home-based programme not reported
	66.6 (41%)	Home exercises with 1–2 planned visits by a local physiotherapist	10 (4:8) lost to follow up
Minns Lowe et al. 2012 [20] UK 2006-2009	Primary TKA	Home-based functional rehabilitation	3, 6 and 12 months
	Osteoarthritis	Home	OKS, KOOS, leg extensor power, timed sit to stand test, 10 metre timed walk
	N = 107 (56:51) received surgery	2 physiotherapist home visits within 2 weeks and at 6–8 weeks after discharge. Assessment of function and rehabilitation progress on gait re-education, and use of walking aids. Twice daily exercise for 3 months: weight, partial knee bends/quarter squats, standing knee flexion and extension wall sits, heel and knee raises, step-overs, and stretches. Task training: getting in and out of a car, getting up from a chair at a table, walking outside and stairs.	46/47 home-based group received 2 visits (98%)
	69.2 (58%)	Controls received usual physiotherapy treatment provided at the hospital without additional home visits	1 (1:0) lost to follow up
Mitchell et al. 2005 [21] UK 1999-2000	Primary unilateral TKR	Home physiotherapy compared with outpatient group provision	12 weeks
	Osteoarthritis	Up to 6 post-discharge home visits by community physiotherapist. Commenced 3–19 days after discharge. Patient assessment and individualised therapy relating to pain relief, knee flexion and extension, gait re-education, home and functional adaptations, reduction of swelling and mobilisation of soft tissues. Before surgery patients received 3 visits.	WOMAC, SF-36, resource use and cost
	N = 115 (57:58)	Controls received exercises and individual treatment 1–2 times a week	Home-based group had a mean of 8.4 sessions. Outpatient group had a mean of 3.5 sessions
	70.3 (57.9%)		1 (0:1) lost to ITT analysis (45 patients withdrawn mainly pre-surgery)
Mockford et al. 2008 [14] Northern Ireland Not specified	Primary TKA	Outpatient physiotherapy	3 months and 1 year
	Osteoarthritis, rheumatoid arthritis	Outpatient department	Oxford Knee Score, SF-12, Bartlett Patella Score, ROM, Walking distance
	N = 143 (71:72)	6 weeks starting within 3 weeks of hospital discharge	Intervention group attended mean 7.3 sessions (range 0–9). 43/71 attended all sessions (61%)
	70.2 (61.5%)	Control received no outpatient physiotherapy following discharge. All patients were given a home exercise regime to follow on discharge	7(4:3) not followed up
Moffet et al. 2004 [18] Canada 1997-1999	Primary TKA	Intensive functional rehabilitation	4, 6, 12 months
	Osteoarthritis	Rehabilitation Institute	WOMAC, SF-36, 6 minute walk test
	N = 77 (38:39)	12 physiotherapist supervised sessions from 2 months after-discharge with individualised home exercises. 60-90mins per week for 6–8 weeks	All intervention patients participated in the 12 sessions
	67.7 (59.7%)	Each session included: warm-up, specific strengthening exercises, functional task-oriented exercises, endurance exercises, and cool-down. ROM, pain and effusion monitored to optimise intervention.	6 (0:6) not followed up at 12 months
		Control group received usual care including possibility of supervised rehabilitation at home	
		All patients were taught a home exercise programme before hospital discharge.	
Monticone et al. 2013 [16] Italy 2010	Primary TKR, osteoarthritis	Home-based functional exercise programme	6 and 12 months
	N = 110 (55:55)	Home	Knee injury and Osteoarthritis Outcome Score (KOOS), Tampa Scale for Kinesiophobia, NRS pain, SF-36
	67 (64%)	Continuation of functional exercises provided in hospital. Cognitive behavioural intervention with home exercise book about the fear-avoidance model and management of kinesiophobia. Monthly phone calls to reinforce adherence.	No patients dropped out of study but no information collected on patient adherence
		Commenced after discharge from rehabilitation unit	0 losses to follow up
		Twice-weekly 60-minute sessions for 6 months	
		No physiotherapy. Advice to stay active	
Piqueras et al. 2013 [22] Spain 2008-2010	Primary TKR, able to walk and with no contra-indications for rehabilitation	Outpatient and home-based telerehabilitation	2 weeks after intervention and 3 months
	Osteoarthritis	5 sessions under therapist supervision at rehabilitation department and 5 sessions at home	ROM, isometric hamstring and quadriceps strength, pain, WOMAC, timed up and go test
	N = 142 (72:70). 181 randomised but 142 completed baseline measures	Commenced after 2 week rehabilitation programme after hospital discharge	18/72 home-based (25%) and 21/70 outpatient (30%) dropped out during first 5 sessions.
	73.5 (72.4%)	Interactive virtual telerehabilitation. Patients received information needed to perform exercises and remote therapist monitoring. Therapy modified as rehabilitation evolved. System used wireless movement sensors, interactive software and a touch-screen computer, and a web-portal.	9 (4:5) lost to follow up
		Daily 1 hour sessions for 10 days	
		Conventional out-patient physical therapy. All randomised patients received a 2 week rehabilitation programme immediately after hospital discharge	
Piva et al. 2010 [26] USA 2007-2008	Unilateral TKR in the last 2-6months	Balance exercises (additional to supervised functional training programme)	2 months and 6 months
	Not specified	Outpatient physical therapy department	WOMAC, Lower Extremity Functional Scale, timed chair rise test, self-selected gait speed over 4 m
	N = 43 (21:22)	Additional balance exercises (agility and perturbation)	84% completed programmes. 64-67% of prescribed exercises completed
	68.5 (71.4%)	Control group received a supervised functional training program without additional balance exercises	8 (3:5) not followed up
		Commenced 2–6 months after surgery	
		All patients received 12 sessions of functional training over 6 weeks	
		Home exercises given to both groups at the end of the supervised programme	
Rajan et al. 2004 et al. [15] UK 1998-1999	Primary TKA	Outpatient physiotherapy	3 months, 6 months and 1 year
	Monoarticular arthrosis	Outpatient	ROM
	N = 120 (59:61)	Average 4–6 physiotherapy sessions	No information on patient adherence
	68.5 (62.9%)	Commenced after discharge from hospital	4 (3:1) not followed up
		Control group did not receive outpatient physiotherapy	
		All patients given a home exercise regime on discharge	
Tousignant et al. 2011 [23] Canada Not specified	TKA	Functional rehabilitation	4 months
	Not specified	Home	Knee range of motion, Berg balance scale, 30 second chair-stand test, WOMAC, Timed up and go, Tinetti test, functional autonomy measu(SMAF), SF-36
	N = 48 (24:24)	Intervention group received tele-rehabilitation through high speed internet. Progressive exercises to reduce disability and improve function in ADL. Family member or friend present to ensure safety	No information on adherence
	66 (unreported)	2 sessions per week for 8 weeks	7 (3:4) not followed up
		Commenced within 5 days of hospital discharge	
		Approx 1 hour duration	
		Control group received usual home care services and outpatient rehabilitation over 2 month period	

### Intervention focus

The focus of the intervention was: movement and exercise [14-16], exercises aimed at managing kinesophobia [17], functional [18,19] or strengthening exercise [20], compared with minimal physiotherapy exercise in seven studies; home compared with outpatient provision in six studies [21-26]; physiotherapy with additional balance [27,28] or cycling components [29] compared with standard physiotherapy in three studies; walking skills compared with more general physiotherapy in one study [30]; and pool-based compared with gym-based provision in one study [31]. Interventions commenced within 6 months of surgery and in the majority of studies within 2 months.

### Patient adherence

Where information was available, patient adherence to the intervention was good with 60% or more of patients completing programmes.

### Outcome measures

Outcomes reported in studies were classified as: patient reported physical function or pain; physiological tests; physical performance tests; and generic health related quality of life measures. The most frequently used physiological outcome was knee range of motion (ROM) expressed as extension and/or flexion in 10 studies [14-16,18,20,23,25,28,30,31]. Less frequently reported outcomes were isometric muscle strength, leg power, and knee oedema. Performance measures reported were walking (walking speed, metres walked in specified time, and time to walk a specified distance), stair ascent and descent tests, and chair rise tests. The 6-minute walk test was the most frequently reported test of walking performance reported in 4 studies.

### Study quality

We completed a risk of bias assessment for each study and summarised these in Table 2. The main potential source of bias was from large and uneven losses to follow up in six studies. Two further studies were judged to be of reasonable quality with overall losses to follow up between 10 and 20%. There was no suggestion of risk of bias in nine studies. There was no clear evidence of publication bias from inspection of funnel plots. However numbers of studies were small for several outcomes and in sub-group analyses.

**Table 2:** **Cochrane risk of bias table**

	**Random sequence generation (selection bias)**	**Allocation concealment (selection bias)**	**Blinding of outcome assessment (detection bias) (patient-reported outcomes)**	**Incomplete outcome data addressed (attrition bias)**	**Lack of selective reporting (reporting bias)**	**Lack of other sources of bias**	**Our evaluation**
Bruun-Olsen et al. 2013 [29]	Yes	Yes	Yes	Yes. 6 (2:4) not followed up	Yes	Yes	Good
Evgeniadis et al. 2008 [19]	Yes	Yes	Yes	Uneven ITT loss to follow up (37.5% intervention and 20% control)	Yes	Yes	Possible bias due to large and uneven losses to follow up
Frost et al. 2002 [17]	Yes	Not clear	Yes	Uneven loss to follow up (intervention 30%, control 54%)	Yes	Yes	Possible bias due to large and uneven losses to follow up
Fung et al. 2012 [27]	Yes	Yes	Yes	Yes	Yes	Yes	Good
Harmer et al. 2009 [30]	Yes	Yes	Yes (mainly)	Yes. ITT, small losses to follow up	Yes	Yes	Good
Kauppila et al. 2010 [13]	Yes	Probably adequate	No	Yes. Losses to follow up: intervention 18%; control 7%. However patients with incomplete data included in authors’ analyses	Yes	Baseline differences in prevalence of comorbidities and WOMAC score.	Possible risk of bias due to uneven losses to follow up
Kramer et al. 2003 [25]. Also data from Minns Lowe 2007 [8]	Not described	Not described	Yes	“Medical issue” losses to follow up differed between groups (7.5% in clinic and 15% in home-based groups)	Yes	Yes. ITT analysis reported as well as per-protocol	Possible risk of bias due to uneven losses to follow up between groups
Liebs et al. 2010 [28]	Yes	Yes	Yes	11.8% intervention and 18.9% control patients lost to 3 month follow up	Yes	Yes	Possible risk of bias due to uneven losses to follow up
Madsen et al. 2013 [24]	Yes	Yes	Yes	10% intervention and 20% control group lost to follow up. Analysis of change scores	Yes	Yes	Possible risk of bias due to uneven losses to follow up
Minns Lowe et al. 2012 [20]	Yes	Yes	Yes	Yes, low losses to follow up at 12 months	Yes	Yes	Good
Mitchell et al. 2005 [21]	Yes	Yes	Self-completed questionnaires	Yes	Yes	Randomisation before surgery with pre-surgical intervention component. Surgery cancelled for 24 patients	Good
Mockford et al. 2008 [14]	Yes	Yes	Yes	4.7% patients excluded from analysis as lost to follow up	Yes	Yes	Good
Moffet et al. 2004 [18]	Yes	Yes	Yes	Yes. Uneven loss to follow up at 12 months (intervention 0%, control 20.5%)	Yes	Yes	Good Possible risk of bias for 12 month outcomes
Monticone et al. 2013 [16]	Yes	Yes	Yes	Yes	Yes	Yes	Good
Piqueras et al. 2013 [22]	Yes	Yes	Yes	Yes	Yes	Yes	Good
Piva et al. 2010 [26]	Yes	Yes	Yes	22.7% control and 14.3% intervention patients lost to follow up	Yes	Yes	Reasonable
Rajan et al. 2004 [15]	Yes	Not described	Yes	5.1% intervention and 1.6% control patients lost to follow up	Yes	Yes	Good
Tousignant et al. 2011 [23]	Yes	Yes	Yes	Similar losses to follow up between groups (intervention 12.5%, control 16.7%)	Yes	3/24 randomised to control withdrew due to knowledge of group allocation	Reasonable

### Comparison of different physiotherapy interventions

Results for comparisons of physiotherapy exercise and no intervention and home-based and outpatient delivery are summarised in Table 3. Meta-analyses used random effects models, an a priori decision based on the known variation in physiotherapy exercise content. Pooled effect sizes are standardised mean differences except for range of motion where mean differences are reported. For the other interventions we provide a brief narrative summary of outcomes.

**Table 3:** **Meta-analyses**

	**Studies**	**Patients**	**Pooled effect size (CI)**	**P-value**	**I** ^**2**^ **(%)**
**Physiotherapy exercise compared with minimal intervention**					
***Physical function***					
3-4 months follow up	3	254	−0.37 [−0.62, −0.12]	0.004	0%
6 month follow up	3	260	−0.43 [−0.95, 0.08]	0.10	76%
12 month follow up	4	397	−0.21 [−0.70, 0.29]	0.42	83%
***Physical function in studies with low risk of bias***					
3-4 months follow up	2	119	−0.35 [−0.62, −0.08]	0.01	0%
6 month follow up	2	185	−0.64 [−1.15, −0.13]	0.01	65%
12 month follow up	2	253	−0.37 [−1.36, 0.61]	0.46	93%
***Pain***					
3-4 months follow up	2	103	−0.45 [−0.85, −0.06]	0.02	0%
6 month follow up	4	287	−0.29 [−0.68, 0.10]	0.15	60%
12 month follow up	4	281	−0.15 [−0.64, 0.35]	0.57	75%
***Pain in studies with low risk of bias***					
3-4 months follow up	1	27	−0.27 [−1.05, 0.50]	0.49	
6 month follow up	2	185	−0.58 [−0.88, −0.29]	0.0001	0%
12 month follow up	1	110	−0.73 [−1.12, −0.35]	0.0002	
***Range of motion extension***					
3-4 months follow up	2	178	−4.14 [−7.10, 1.18]	0.006	82%
6 month follow up	1	74	0.00 [−1.37, 1.37]	1.00	
12 month follow up	2	217	0.42 [−0.54, 1.38]	0.39	0%
***Range of motion extension in studies with low risk of bias***					
3-4 months follow up	1	143	−2.60 [−4.48, −0.72]	0.007	
6 month follow up	0				
12 month follow up	1	143	0.20 [−0.92, 1.32]	0.73	
***Range of motion flexion***					
3-4 months follow up	4	321	−5.23 [−11.16, 0.70]	0.08	83%
6 month follow up	3	217	−4.06 [−6.67, −1.46]	0.02	0%
12 month follow up	4	360	−2.21 [−4.31, −0.10]	0.04	0%
***Range of motion flexion in studies with low risk of bias***					
3-4 months follow up	1	116	−2.00 [−4.78, 0.78]	0.16	
6 month follow up	1	116	−5.00 [−8.14, −1.86]	0.002	
12 month follow up	2	259	−2.38 [−4.80, 0.05]	0.05	0%
***Walking***					
Longest follow up (all 12 months)	3	169	−0.17 [−0.48, 0.13]	0.27	0%
**Home-based compared with outpatient delivery of physiotherapy exercise**					
***Physical function***					
3-4 months follow up	4	310	−0.03 [−0.25, 0.19]	0.80	0%
6 month follow up	2	150	0.05 [−0.27, 0.38]	0.74	0%
12 month follow up	2	214	0.11 [−0.16, 0.38]	0.42	0%
***Physical function in studies with low risk of bias***					
3-4 months follow up	2	199	−0.15 [−0.43, 0.13]	0.29	0%
6 month follow up	1	82	0.18 [−0.25, 0.62]	0.41	
12 month follow up	1	87	0.01 [−0.41, 0.44]	0.95	
***Pain***					
3-4 months follow up	3	248	−0.00 [−0.25, 0.25]	0.98	0%
6 month follow up	1	85	−0.05 [−0.48, 0.38]	0.82	
12 month follow up	1	92	−0.13 [−0.53, 0.28]	0.55	
***Pain in studies with low risk of bias***					
3-4 months follow up	2	207	−0.07 [−0.35, 0.20]	0.59	0%
6 month follow up	1	85	−0.05 [−0.48, 0.38]	0.82	
12 month follow up	1	92	−0.13 [−0.53, 0.28]	0.55	
***Range of motion extension***					
3-4 months follow up	3	261	−0.21 [−0.46, 0.05]	0.11	6%
6 month follow up	0				
12 month follow up	1	83	−0.18 [−0.61, 0.25]	0.41	
***Range of motion extension in studies with low risk of bias***					
3-4 months follow up	3	261	−0.21 [−0.46, 0.05]	0.11	6%
6 month follow up	0				
12 month follow up	1	83	−0.18 [−0.61, 0.25]	0.41	
***Range of motion flexion***					
3-4 months follow up	3	329	−0.22 [−0.44, −0.01]	0.04	0%
6 month follow up	1	68	−0.18 [−0.65, 0.30]	0.47	
12 month follow up	2	202	0.07 [−0.21, 0.35]	0.61	0%
***Range of motion flexion in studies with low risk of bias***					
3-4 months follow up	3	329	−0.22 [−0.44, −0.01]	0.04	0%
6 month follow up	1	68	−0.18 [−0.65, 0.30]	0.47	
12 month follow up	1	83	−0.05 [−0.48, 0.38]	0.81	
***Walking***					
Longest follow up (2 studies 12 months, 1 study 6 months)	3	267	−0.02 [−0.26, 0.22]	0.87	37%

Pooled effect sizes are standardised mean differences except for range of motion where *mean* differences are reported (random effects models).

### Physiotherapy exercise compared with minimal intervention

In seven studies, patients randomised to physiotherapy exercise intervention were compared with a control group receiving no intervention or minimal intervention [14-20]. For control group patients, minimal treatment was either only inpatient rehabilitation common to both groups [20], or instructions on home exercise given before discharge [15-19] or at a two-month post-operative outpatient appointment [14].

#### Patient reported physical function

Results for all intervention comparisons and outcomes are summarised in Table 3.

Data was available at one or more time points for 5 studies that compared a physiotherapy intervention with a control group that received minimal physiotherapy [14,15,17,19,20]. Studies reported Western Ontario and McMaster Universities Arthritis Index (WOMAC) physical function, Oxford Knee Score, Knee Injury and Osteoarthritis Outcome Score (KOOS) activities of daily living scale or Iowa Level of Assistance Scale (ILAS) total score.

**Figure 2:**
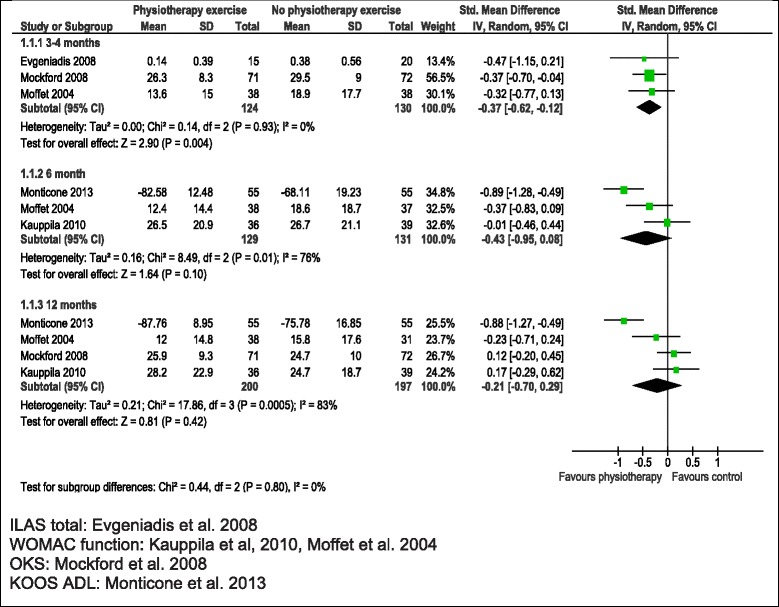
**Physiotherapy exercise compared with no intervention: physical function.**

As shown in the meta-analysis in Table 3 and Figure 2, at 3–4 months, physiotherapy exercise was associated with an improvement in physical function in 3 studies with 254 patients [15,19,20], average SMD −0.37 (95% CI −0.62, −0.12; p = 0.004). At 6 months there was a non-significant trend for benefit in 3 studies [14,17,19], SMD −0.43 (95%CI −0.95, 0.08; p = 0.10), and little difference between groups in 4 studies [14,15,17,19] at 12 months. Heterogeneity was high in studies reporting outcomes at 6 and 12 months and this was not explained by inclusion of studies with high risk of bias [14,20]. After exclusion of these studies, benefit was apparent at both 3 and particularly at 6 months, SMD −0.64 (95% CI −1.15, −0.13; p = 0.01), but included only 2 studies at each follow up.

#### Patient reported pain

**Figure 3:**
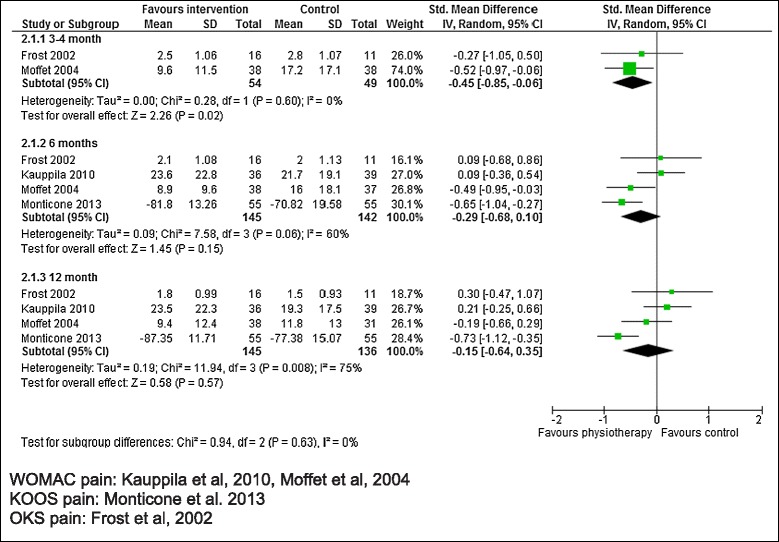
**Physiotherapy exercise compared with no intervention: pain.**

Four studies reported a pain outcome at one or more follow up times [14,17-19]. Studies reported WOMAC pain, KOOS pain or OKS pain. As shown in Table 3 and Figure 3, in two studies with 103 patients [18,19], a pain outcome was reported at 3–4 months with average SMD −0.45 (95% CI −0.85, −0.06; p = 0.02) favouring physiotherapy exercise. There was a trend for benefit at 6 months in 4 studies with 287 patients [14,17,18,32], average SMD −0.29 (95% CI −0.68, 0.10; p = 0.15). At 12 month follow up there was little to suggest benefit for patients receiving physiotherapy exercise compared with untreated controls in 4 studies with 281 patients [14,17,18,32]. Heterogeneity was high at 6 and 12 month follow up. Only one study had low risk of bias at each of 3–4 and 12 months [17] precluding meta-analysis. At 6 months, 2 higher quality studies [17,19] showed benefit, average SMD −0.58 (95% CI −0.88, −0.29; p = 0.0001).

#### Range of motion

**Figure 4:**
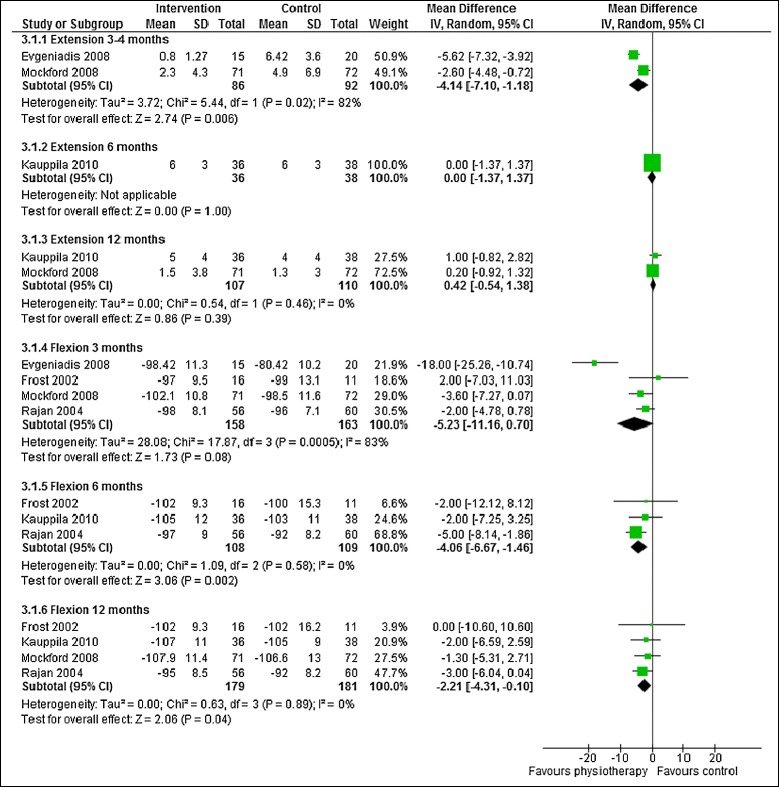
**Physiotherapy exercise compared with no intervention: ROM.**

ROM extension data suitable for meta-analysis was available from 3 studies with 252 patients [14,15,20], and ROM flexion from 5 studies with 396 patients [14-16,18,20]. As shown in Table 3 and Figure 4, there was little to suggest long-term benefit for outpatient physiotherapy improved long-term ROM extension. Benefit was only evident in 2 studies with follow up at 3 months after total knee replacement. For ROM flexion there was evidence of improved flexion in patients receiving physiotherapy exercise, particularly after 6 and 12 months. Benefit was seen in studies with low risk of bias but this was based on a small number of studies.

#### Physical performance

Measures of walking performance (metres walked in a set time, time to walk a specified distance and walking speed) were combined with attention paid to direction of effect. An improvement in walking performance in three3 studies with 169 patients [14,18,19] was not significant, average SMD −0.17 (95% CI −0.48, 0.13; p = 0.27). There was no heterogeneity across studies.

### Home-based compared with outpatient delivery of physiotherapy exercise

Home-based provision was compared with outpatient physiotherapy in six studies [21-26].

#### Patient reported physical function

Data was available at one or more time points for five studies comparing the outcomes of home-based physiotherapy exercise with outpatient or standard provision [21,22,24-26].

**Figure 5:**
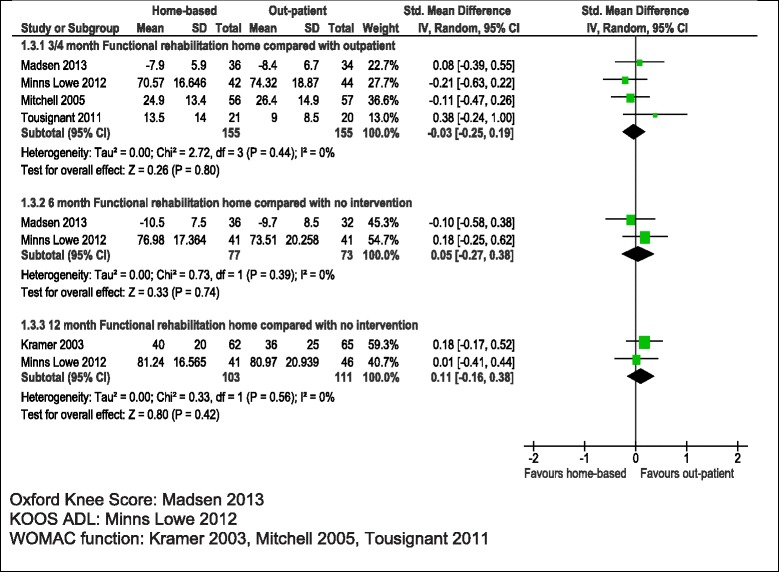
**Home-based compared with outpatient physiotherapy exercise: physical function.**

Physical function was measured using WOMAC, KOOS and OKS in 5 studies with up to 436 patients followed up [21,22,24-26]. As shown in Table 3 and Figure 5, there was no suggestion of a difference in functional outcome between home and outpatient provision at 3–4 months, 6 months or 12 months. For example at 3–4 months, the average SMD was −0.03 (95% CI −0.25, 0.19; p = 0.80). No heterogeneity was apparent and consideration of higher quality studies did not suggest any difference in outcomes after home or outpatient physiotherapy exercise. However numbers of studies to base this on were small.

#### Patient reported pain

**Figure 6:**
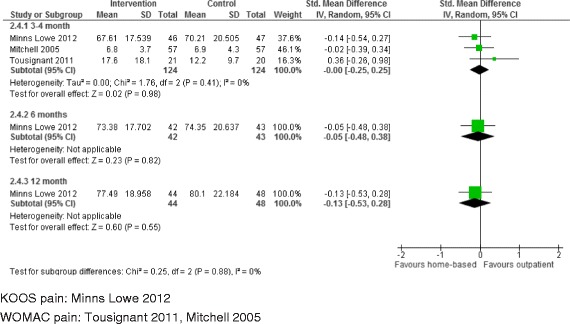
**Home-based compared with outpatient physiotherapy exercise: pain.**

Studies reported WOMAC pain, KOOS pain or VAS pain. Data was available at 3–4 months for three studies with 248 patients [21,22,24]. As shown in Table 3 and Figure 6, there was no difference in pain outcome between patients randomised to home-based or outpatient physiotherapy exercise, average SMD −0.00 (95% CI −0.25, 0.25;p = 0.98). One study followed up 85 and 92 patients at 6 and 12 months [21] and showed no benefit for reduced pain at either follow up.

#### Range of motion

**Figure 7:**
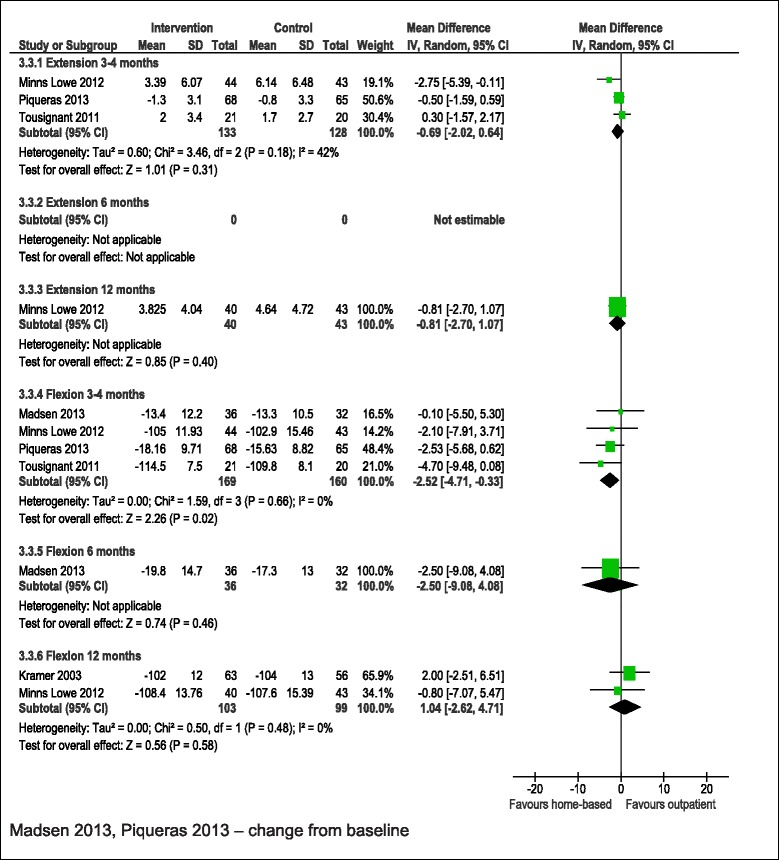
**Home-based compared with outpatient physiotherapy exercise: ROM.**

ROM extension was reported in 3 studies with 261 patients [21,23,24] and ROM flexion in five studies with 448 patients [21,23-26]. Outcomes are summarised in Table 3 and Figure 7. There was no suggestion of a difference in ROM extension between randomised groups at any time point. For ROM flexion there was an improved ROM flexion at 3–4 months in patients who received home-based physiotherapy exercise compared with outpatient provision [21,23-25]. This was maintained in studies with low risk of bias [21,23,24]. There was no evidence for longer term benefit in 2 studies [21,26].

#### Physical performance

In 3 studies with 267 patients randomised [21,25,26] there was no suggestion that walking performance differed between groups.

### Pool-based physiotherapy

One study compared pool-based physiotherapy with gym-based provision [31]. There were no differences between treatments in WOMAC physical function, WOMAC pain or ROM extension and flexion at the end of the interventions and at 26 week follow up.

### Walking skills

In one study a walking skills programme was provided from 6 weeks after surgery for 6–8 weeks. A comparison group received “usual physiotherapy care”. All patients previously received extensive physiotherapy after surgery at a rehabilitation centre and subsequently in an outpatient setting [30]. There were no statistically significant differences in KOOS outcomes or ROM between groups at 9 months. However a difference in the 6 minute walk test favouring the walking skills group noted immediately after the intervention was sustained at 9 months.

### Additional physiotherapy components

One study with 159 patients evaluated addition of ergometer cycling to a general physiotherapy intervention [29]. There were no differences in pain outcome between randomised groups at any of the follow up intervals from 3–4 months to 24 months.

Two studies evaluated addition of a balancing component to a general physiotherapy intervention with a total of 93 patients randomised [27,28]. Studies reported different follow up times but individually there was no evidence for improvement in LEFS or WOMAC physical function. Similarly, NRS pain and WOMAC pain were similar at all follow up periods. One study which included additional balance training reported ROM extension and flexion at short term follow up [28]. There were no differences in either measure between intervention and control groups.

## Discussion

Randomised controlled trials of physiotherapy and exercise interventions after total knee replacement provide some evidence for-short term effectiveness. In the key analysis comparing patients who received a programme of physiotherapy exercise with those receiving no intervention there were short-term benefits for physical function, SMD −0.37 (95% CI −0.62, −0.12; p = 0.004), and pain, SMD −0.45 (95% CI −0.85, −0.06; p = 0.02). However, these small to medium sized effects [33], were based on only 3 studies with 254 patients, and 2 studies with 103 patients randomised respectively. No benefit was apparent regarding longer-term improvements to function and pain in the randomised controlled trials of physiotherapy exercise that we identified. For physical function this observation was based on 4 studies with high heterogeneity which was not explained by consideration of the 2 studies with low risk of bias.

With a more robust evidence base this could be interpreted as a speeding up of recovery attributable to physiotherapy exercise but with a similar long-term level of recovery irrespective of post-discharge care. More realistically it suggests the need for appropriately powered studies.

There is no up-to-date national guidance to support the facilitation of early recovery using exercise-based rehabilitation. Physiotherapy should also address patient expectations [34,35], since the key expectations of patients undergoing knee replacement relate to long-term functional and pain outcomes [36-39]. Strategies to improve communication and provide patients with a better understanding of realistic expectations after knee replacement need to be considered prior to surgery [35].

The problems of poor medium to long-term patient outcomes after total knee replacement are recognised. Judge and colleagues assessed functional improvement according to a number of success criteria and concluded that 14–36% of patients did not improve or were worse 12 months after surgery [40]. In a study of patients with moderate to severe hip or knee arthritis, Hawker and colleagues reported that only about 50% of patients had a clinically important improvement in WOMAC score at a median of 16 months after surgery [41]. Regarding post-surgical pain [42], unfavourable outcomes were reported by 10 to 34% of knee replacement patients in 11 representative populations identified by Beswick and colleagues. There is clearly a need for rehabilitation strategies that can enhance recovery for the majority of patients and target patients whose post-surgical experience is unfavourable. Westby and Backman highlighted the importance of utilising strategies to empower patients in the rehabilitation process [35]. Provision of tailored rehabilitation programmes may assist in maximising individual outcome after surgery and are worthy of further research.

Knee range of motion is commonly measured after knee replacement and is a component of clinician-based outcome measures such as the Knee Society Clinical Rating System [43]. Across the trials reporting range of motion, we observed benefit for physiotherapy exercise in studies with low risk of bias compared with controls for flexion only. However, although useful as a trial outcome [16], ROM is considered a poor marker of implant success [44,45], and may not influence patient satisfaction with their replacement [46]. As with all the results of our meta-analyses, conclusions are limited by the small number of small studies that we identified.

The need for measures of both gait and a patient reported functional outcome is recognised [47,48]. A measure of walking performance was included in over half of the studies we identified but we were unable to identify any benefit from physiotherapy exercise. In four higher quality studies there was a trend for benefit but this was not statistically significant.

Studies of physiotherapy exercise after hospital discharge are pragmatic in nature with patients who have consented to be randomised free to participate to whatever extent they choose or to seek physiotherapy exercise additional to that in their allocated group. When reported, uptake and adherence by patients randomised to groups with a specific physiotherapy exercise intervention was good. A limitation of the review is the possibility that patients in the minimally treated control group received some physiotherapy. We did not anticipate that being allocated to a control group would preclude the possibility of referral for physiotherapy on the basis of individual clinical need. For example, Moffet and colleagues reported that about a quarter of control group patients received some home physiotherapy service [19]. However, in the subgroup of studies comparing physiotherapy exercise provision with minimal provision there was little to suggest overlap with the subgroups comparing alternative methods of provision.

There were insufficient studies with adequate patient numbers to provide conclusive evidence on different methods of provision. Physiotherapy exercise provided at home is an appealing approach with the possibility of wider acceptability and uptake. However, equivalence or non-inferiority trials need large numbers of patients and have yet to be undertaken. Our meta-analysis included only 310 patients for the short-term physical function outcome and less for the key longer-term outcomes. Similar issues of study size affect interpretation of physiotherapy exercise provided in a hydrotherapy pool, enhanced with additional cycling and balancing components, or focusing on walking skills. This highlights the difficulty of developing a complex physiotherapy exercise intervention.

A search for ongoing trials in ClinicalTrials.gov identified some randomised trials of physiotherapy exercise in total knee replacement. These are evaluating the effect of additional strength training [49,50], independent exercise prescription compared with supervised exercise [51], and progressive resistance rehabilitation compared with traditional rehabilitation [52]. One ongoing study will evaluate intensive physiotherapy for patients performing poorly at 6 weeks following total knee replacement [53]. Targeting physiotherapy at those with greatest functional need may be a valuable approach but many other patients have sub-optimal outcomes [54], and may also benefit from appropriate intervention.

An important problem that home-based physiotherapy exercise may address is that uptake of rehabilitation is frequently low and that patients who do not attend are more likely to be those with poorer functional health. Optimising uptake and adherence to interventions is an important issue in rehabilitation [55,56]. In their systematic review of interventions for enhancing adherence with physiotherapy, McLean and colleagues found only short-term evidence of effectiveness of exercise adherence strategies and little evidence that home based-interventions are associated with good adherence [55]. They concluded that a strategy to improve adherence to physiotherapy treatment should probably be multi-dimensional.

Despite the inclusion of 18 randomised controlled trials compared with 6 trials in the review of Minns Lowe and colleagues, our conclusion is similar with a possible short-term benefit for physiotherapy exercise after knee replacement. There was only limited evidence from a single small study focusing on walking skills to suggest that any benefit was maintained at longer-term follow up. We concur with Minns Lowe and colleagues and Muller and colleagues [12] that further research is needed.

Some physiotherapy exercise will generally be provided to patients with total knee replacement even if this only comprises advice following on from inpatient rehabilitation. Healthcare professionals and policy makers need to know what content and duration of physiotherapy exercise is necessary to improve short and long-term outcomes and which patients are likely to benefit. Appropriate care can then be provided to each individual patient. Future studies should include credible evaluation of methods with well-designed and appropriately powered randomised trials with a focus on completeness of follow up.

## Conclusion

After recent primary total knee replacement, physiotherapy exercise interventions show short-term improvements in physical function. However this conclusion is based on meta-analysis of a few small studies and no long-term benefits of physiotherapy or exercise intervention were identified.

## Additional files

Additional file 1:
**Reasons for exclusion.**
Additional file 2:
**Reasons for exclusion references.**

## Abbreviations

SMD: Standardised mean differences
MD: Mean differences
WOMAC: Western Ontario and McMaster Universities arthritis index
KOOS: Knee injury and osteoarthritis outcome score
ILAS: Iowa level of assistance scale
ROM: Range of motion
OKS: Oxford Knee Score
VAS: Visual analogue scale
NRS: Numerical rating scale
