# Supplementary material for: Effectiveness of physiotherapy exercise following total knee replacement: systematic review and meta-analysis
Source: BMC Musculoskelet Disord. 2015 Feb 7;16:15. doi: 10.1186/s12891-015-0469-6 (PMC4333167; doi:10.1186/s12891-015-0469-6)
Supplement: Additional file 1: — Reasons for exclusion. [file 12891_2015_469_MOESM1_ESM.docx]

**Appendix 1. Reasons for exclusion**

**Acupuncture:** Tsang 2007[1]

**Continuous passive motion:** Alkire 2010 [2], Aubriot 1993 [3], Beaupre 2001 [4], Bennett 2005 [5], Bruun-Olsen 2009 [6], Chen 2000 [7], Chiarello 1997 [8], Davies 2003 [9], Denis 2006 [10], Harms 1991 [11], Huang 2003 [12], Jordan 1995 [13], Kim 2009 [14], Lenssen 2008 [15], Sewell 2007 [16], Ververeli 1995 [17], Wasilewski 1990 [18]

**Electrical/ magnetic:** Avramidis 2003 [19], Blasczak 2004 [20], Gotlin 1994 [21], Gremeaux 2008 [22], Petterson 2009 [23], Rockstroh 2010 [24]

**Inpatient:** Beard 2002 [25], Esler 1999 [26], Grissom 2001 [27], Hecht 1983 [28], Horton 2002 [29], Korlarz 1999 [30], Kuiken 2004 [31], Kumar 1996 [32], Lang 1998 [33], Lenssen 2006 [34], Liu 2009 [35], Lysack 2005 [36], Moon 1997 [37], Rahmann 2009 [38], Renkawitz 2010 [39], Scarcella 1995 [40], Zenios 2002 [41]

**Minority TKR:** Bellelli 2010 [42], Bellelli 2009 [43], Bulthuis 2007 [44], Bulthuis 2008 [45], Isakov 2007 [46]

**Not TKR:** Deyle 2000 [47], Gassner 2007 [48], Jenkins 2008 [49], Johnsson 1988 [50], Lamb 2008 [51], Pisters 2010 [52], Wang 2007 [53]

**Not RCT:** Codine 2004 [54], Coulter 2009 [55], Giaquinto 2007 [56], Ulreich 2007 [57], Ulrich 2007 [58]

**Pre-surgery:** Beaupre 2004 [59], Crowe 2003 [60], D’Lima 1996 [61], Gill 2009 [62], Liebergall 1999 [63], Ling 2008 [64], Nunez 2006 [65], Rooks 2006 [66], Soni 2010 [67], Taverner 2010 [68], Topp 2009 [69], Weidenhielm 1993 [70], Williamson 2007 [71]

**Protocol only:** Lin 2009 [72]

**Retrospective identification:** Lastayo 2009 [73], Trudelle-Jackson 2004 [74], Valtonen 2010 [75]
