# Supplementary material for: Effectiveness of physiotherapy exercise following total knee replacement: systematic review and meta-analysis
Source: BMC Musculoskelet Disord. 2015 Feb 7;16:15. doi: 10.1186/s12891-015-0469-6 (PMC4333167; doi:10.1186/s12891-015-0469-6)
Supplement: Additional file 2: — Reasons for exclusion references. [file 12891_2015_469_MOESM2_ESM.docx]

**Appendix 2. Reasons for exclusion references**

1. Tsang RC-C, Tsang P-L, Ko C-Y, Kong BC-H, Lee W-Y, Yip H-T: Effects of acupuncture and sham acupuncture in addition to physiotherapy in patients undergoing bilateral total knee arthroplasty-a randomized controlled trial. ***Clin Rehab*** 2007, 21:719-728.

2. Alkire MR, Swank ML: Use of inpatient continuous passive motion versus no CPM in computer-assisted total knee arthroplasty. ***Orthop Nurs*** 2010, 29:36-40.

3. Aubriot JH, Guincestre JY, Grandbastien B: Value of continuous passive motion in the early rehabilitation of total knee arthroplasty. Prospective study apropos of 120 medical records. ***Revue de Chirurgie Orthopedique et Reparatrice de l Appareil Moteur*** 1993, 79:586-590.

4. Beaupre LA, Davies DM, Jones CA, Cinats JG: Exercise combined with continuous passive motion or slider board therapy compared with exercise only: A randomized controlled trial of patients following total knee arthroplasty. ***Phys Ther*** 2001, 81:1029-1037.

5. Bennett LA, Brearley SC, Hart JAL, Bailey MJ: A comparison of 2 continuous passive motion protocols after total knee arthroplasty: a controlled and randomized study. ***J Arthroplasty*** 2005, 20:225-233.

6. Bruun-Olsen V, Heiberg KE, Mengshoel AM: Continuous passive motion as an adjunct to active exercises in early rehabilitation following total knee arthroplasty - a randomized controlled trial. ***Disabil Rehab*** 2009, 31:277-283.

7. Chen B, Zimmerman JR, Soulen L, DeLisa JA: Continuous passive motion after total knee arthroplasty: a prospective study. ***Am J Phys Med Rehab*** 2000, 79:421-426.

8. Chiarello CM, Gundersen L, O'Halloran T: The effect of continuous passive motion duration and increment on range of motion in total knee arthroplasty patients. ***J Orthop Sports Phys*** 1997, 25:119-127.

9. Davies DM, Johnston DWC, Beaupre LA, Lier DA: Effect of adjunctive range-of-motion therapy after primary total knee arthroplasty on the use of health services after hospital discharge. ***Canadian J Surg*** 2003, 46:30-36.

10. Denis M, Moffet H, Caron F, Ouellet D, Paquet J, Nolet L: Effectiveness of continuous passive motion and conventional physical therapy after total knee arthroplasty: A randomized clinical trial. ***Phys Ther*** 2006, 86:174-185.

11. Harms M, Engstrom B: Continuous passive motion as an adjunct to treatment in the physiotherapy management of the total knee arthroplasty patient. ***Physiotherapy*** 1991, 77:301-307.

12. Huang D, Peng Y, Su P, Ye W, Liang A: The effect of continuous passive motion after total knee arthroplasty on joint function. ***Chinese Journal of Clinical Rehabilitation*** 2003, 7:1661-1662.

13. Jordan LR, Siegel JL, Olivo JL: Early flexion routine: An alternative method of continuous passive motion. ***Clin Orthop Relat Res*** 1995:231-233.

14. Kim TK, Park KK, Yoon SW, Kim SJ, Chang CB, Seong SC: Clinical value of regular passive ROM exercise by a physical therapist after total knee arthroplasty. ***Knee Surg Sport Tr A*** 2009, 17:1152-1158.

15. Lenssen TAF, Van Steyn MJA, Crijns YHF, Waltje EMH, Roox GM, Geesink RJT, Brandt PAVD, De Bie RA: Effectiveness of prolonged use of continuous passive motion (CPM), as an adjunct to physiotherapy, after total knee arthroplasty. ***BMC Musculoskelet Disord*** 2008, 9.

16. Sewell MD, Ang SC: Use of continuous passive motion after primary knee arthroplasty. ***Ann Royal Coll Surg*** 2007, 89:825.

17. Ververeli PA, Sutton DC, Hearn SL, Booth Jr RE, Hozack WJ, Rothman RR: Continuous passive motion after total knee arthroplasty: Analysis of cost and benefits. ***Clin Orthop Relat Res*** 1995:208-215.

18. Wasilewski SA, Woods LC, Torgerson WR, Jr., Healy WL: Value of continuous passive motion in total knee arthroplasty. ***Orthopedics*** 1990, 13:291-295.

19. Avramidis K, Strike PW, Taylor PN, Swain ID: Effectiveness of electric stimulation of the vastus medialis muscle in the rehabilitation of patients after total knee arthroplasty. ***Arch Phys Med Rehabil*** 2003, 84:1850-1853.

20. Blasczak E, Franek A, Klimczak J: Early results of rehabilitation after endoprothesis plasty of knee joints. ***Polski Merkuriusz Lekarski*** 2004, 17:474-478.

21. Gotlin RS, Hershkowitz S, Juris PM, Gonzalez EG, Scott WN, Insall JN: Electrical stimulation effect on extensor lag and length of hospital stay after total knee arthroplasty. ***Arch Phys Med Rehabil*** 1994, 75:957-959.

22. Gremeaux V, Renault J, Pardon L, Deley G, Lepers R, Casillas J-M: Low-frequency electric muscle stimulation combined with physical therapy after total hip arthroplasty for hip osteoarthritis in elderly patients: a randomized controlled trial. ***Arch Phys Med Rehabil*** 2008, 89:2265-2273.

23. Petterson SC, Mizner RL, Stevens JE, Raisis LEO, Bodenstab A, Newcomb W, Snyder-Mackler L: Improved function from progressive strengthening interventions after total knee arthroplasty: A randomized clinical trial with an imbedded prospective cohort. ***Arthritis Care Res*** 2009, 61:174-183.

24. Rockstroh G, Schleicher W, Krummenauer F: Effectiveness of microcurrent therapy as a constituent of post-hospital rehabilitative treatment in patients after total knee alloarthroplasty - a randomized clinical trial. ***Rehabilitation*** 2010, 49:173-179.

25. Beard DJ, Murray DW, Rees JL, Price AJ, Dodd CAF: Accelerated recovery for unicompartmental knee replacement--a feasibility study. ***Knee*** 2002, 9:221-224.

26. Esler CN, Lock K, Harper WM, Gregg PJ: Manipulation of total knee replacements. Is the flexion gained retained? ***J Bone Joint Surg Br*** 1999, 81:27-29.

27. Grissom SP, Dunagan L: Improved satisfaction during inpatient rehabilitation after hip and knee arthroplasty: A retrospective analysis. ***Am J Phys Med Rehabil*** 2001, 80:798-803.

28. Hecht PJ, Bachmann S, Booth RE, Jr., Rothman RH: Effects of thermal therapy on rehabilitation after total knee arthroplasty. A prospective randomized study. ***Clin Orthop Relat Res*** 1983:198-201.

29. Horton TC, Jackson R, Mohan N, Hambidge JE: Is routine splintage following primary total knee replacement necessary? A prospective randomised trial. ***Knee*** 2002, 9:229-231.

30. Kolarz G, Scherak O, El Shohoumi M, Wottawa A: Results of inpatient rehabilitation measures after implantation of knee endoprotheses - Results after one year. ***Aktuelle Rheumatologie*** 1999, 24:22-26.

31. Kuiken TA, Amir H, Scheidt RA: Computerized biofeedback knee goniometer: acceptance and effect on exercise behavior in post-total knee arthroplasty rehabilitation. ***Arch Phys Med Rehabil*** 2004, 85:1026-1030.

32. Kumar PJ, McPherson EJ, Dorr LD, Wan Z, Baldwin K: Rehabilitation after total knee arthroplasty: a comparison of 2 rehabilitation techniques. ***Clin Orthop Relat Res*** 1996:93-101.

33. Lang CE: Comparison of 6- and 7-day physical therapy coverage on length of stay and discharge outcome for individuals with total hip and knee arthroplasty. ***J Orthop Sports Phys Ther*** 1998, 28:15-22.

34. Lenssen AF, Crijns YHF, Waltje EMH, Van Steyn MJA, Geesink RJT, Van Den Brandt PA, De Bie RA: Efficiency of immediate postoperative inpatient physical therapy following total knee arthroplasty: An RCT. ***BMC Musculoskelet Disord*** 2006, 7.

35. Liu P, Li L, Zhang YK, Li M, Kane K, Wang YH, Lin JX, Ding M, Wang SF, Zhou L *et al*: A comparison of two rehabilitation protocols after simultaneous bilateral total knee arthroplasty: A controlled, randomized study. ***J Int Med Res*** 2009, 37:746-756.

36. Lysack C, Dama M, Neufeld S, Andreassi E: A compliance and satisfaction with home exercise: a comparison of computer-assisted video instruction and routine rehabilitation practice. ***Journal of Allied Health*** 2005, 34:76-82.

37. Moon MS, Kim JM, Woo YK: Restoration of knee motion after total knee arthroplasty: subvastus approach and alternate flexion and extension splintage. ***Ryumachi*** 1997, 37:146.

38. Rahmann AE, Brauer SG, Nitz JC: A specific inpatient aquatic physiotherapy program improves strength after total hip or knee replacement surgery: a randomized controlled trial. ***Arch Phys Med Rehabil*** 2009, 90:745-755.

39. Renkawitz T, Rieder T, Handel M, Koller M, Drescher J, Bonnlaender G, Grifka J: Comparison of two accelerated clinical pathways--after total knee replacement how fast can we really go? ***Clin Rehabil*** 2010, 24:230-239.

40. Scarcella JB, Cohn BT: The effect of cold therapy on the postoperative course of total hip and knee arthroplasty patients. ***American Journal of Orthopedics*** 1995, 24:847-852.

41. Zenios M, Wykes P, Johnson DS, Clayson AD, Kay P: The use of knee splints after total knee replacements. ***Knee*** 2002, 9:225-228.

42. Bellelli G, Buccino G, Bernardini B, Padovani A, Trabucchi M: Action observation treatment improves recovery of postsurgical orthopedic patients: Evidence for a top-down effect? ***Arch Phys Med Rehabil*** 2010, 91:1489-1494.

43. Bellelli G, Buccino G, Garrah M, Padovani A, Trabucchi M: Action observation treatment in the rehabilitation of post-surgical orthopaedic patients: A randomised controlled trial. ***Eur J Neurol*** 2009, 16 (S3):308.

44. Bulthuis Y, Drossaers-Bakker KW, Taal E, Rasker J, Oostveen J, van't Pad Bosch P, Oosterveld F, van de Laar M: Arthritis patients show long-term benefits from 3 weeks intensive exercise training directly following hospital discharge. ***Rheumatology*** 2007, 46:1712-1717.

45. Bulthuis Y, Mohammad S, Braakman-Jansen LMA, Drossaers-Bakker KW, van de Laar MAFJ: Cost-effectiveness of intensive exercise therapy directly following hospital discharge in patients with arthritis: results of a randomized controlled clinical trial. ***Arthritis Rheum*** 2008, 59:247-254.

46. Isakov E: Gait rehabilitation: a new biofeedback device for monitoring and enhancing weight-bearing over the affected lower limb. ***Europa Medicophysica*** 2007, 43:21-26.

47. Deyle GD, Henderson NE, Matekel RL, Ryder MG, Garber MB, Allison SC: Effectiveness of manual physical therapy and exercise in osteoarthritis of the knee. A randomized, controlled trial. ***Ann Int Med*** 2000, 132:173-181.

48. Gassner K, Einsiedel T, Linke M, Görlich P, Mayer J: Does mental training improve learning to walk with an above-knee prosthesis?. ***Orthopade*** 2007, 36:673-678.

49. Jenkins C, Barker KL, Pandit H, Dodd CAF, Murray DW: After partial knee replacement, patients can kneel, but they need to be taught to do so: A single-blind randomized controlled trial. ***Phys Ther*** 2008, 88:1012-1021.

50. Johnsson R, Melander A, Onnerfält R: Physiotherapy after total hip replacement for primary arthrosis. ***Scand J Rehab Med*** 1988, 20:43-45.

51. Lamb SE, Toye F, Barker KL: Chronic disease management programme in people with severe knee osteoarthritis: efficacy and moderators of response. ***Clin Rehabil*** 2008, 22:169-178.

52. Pisters MF, Veenhof C, Schellevis FG, De Bakker DH, Dekker J: Long-term effectiveness of exercise therapy in patients with osteoarthritis of the hip or knee: A randomized controlled trial comparing two different physical therapy interventions. ***Osteoarthritis Cart*** 2010, 18:1019-1026.

53. Wang T-J, Belza B, Elaine Thompson F, Whitney JD, Bennett K: Effects of aquatic exercise on flexibility, strength and aerobic fitness in adults with osteoarthritis of the hip or knee. ***J Adv Nurs*** 2007, 57:141-152.

54. Codine P, Dellemme Y, Denis-Laroque F, Herisson C: The use of low velocity submaximal eccentric contractions of the hamstring for recovery of full extension after total knee replacement: A randomized controlled study. ***Isokinet Exerc Sci*** 2004, 12:215-218.

55. Coulter CL, Weber JM, Scarvell JM: Group physiotherapy provides similar outcomes for participants after joint replacement surgery as 1-to-1 physiotherapy: a sequential cohort study. ***Arch Phys Med Rehabil*** 2009, 90:1727-1733.

56. Giaquinto S, Ciotola E, Margutti F: Gait during hydrokinesitherapy following total knee arthroplasty. ***Disabil Rehabil*** 2007, 29:737-742.

57. Ulreich A, Kullich W, Klein G, Ramach W: Results of a multidisciplinary rehabilitation concept after total knee replacement. ***Aktuelle Rheumatologie*** 1997, 22:211-216.

58. Ulrich SD, Bhave A, Marker DR, Seyler TM, Mont MA: Focused rehabilitation treatment of poorly functioning total knee arthroplasties. ***Clin Orthop Relat Res*** 2007, 464:138-145.

59. Beaupre LA, Lier D, Davies DM, Johnston DBC: The effect of a preoperative exercise and education program on functional recovery, health related quality of life, and health service utilization following primary total knee arthroplasty. ***J Rheumatol*** 2004, 31:1166-1173.

60. Crowe J, Henderson J: Pre-arthroplasty rehabilitation is effective in reducing hospital stay. ***Can J Occup Ther*** 2003, 70:88-96.

61. D'Lima DD, Colwell CW, Jr., Morris BA, Hardwick ME, Kozin F: The effect of preoperative exercise on total knee replacement outcomes. ***Clin Orthop Relat Res*** 1996:174-182.

62. Gill SD, McBurney H, Schulz DL: Land-based versus pool-based exercise for people awaiting joint replacement surgery of the hip or knee: results of a randomized controlled trial. ***Arch Phys Med Rehabil*** 2009, 90:388-394.

63. Liebergall M, Soskolne V, Mattan Y, Feder N, Segal D, Spira S, Schneidman G, Stern Z, Israeli A: Preadmission screening of patients scheduled for hip and knee replacement: impact on length of stay. ***Clinical Performance & Quality Health Care*** 1999, 7:17-22.

64. Ling SM: Does acupuncture improve symptoms in patients with osteoarthritis who are awaiting knee replacement surgery? ***Nat Clin Pract Rheum*** 2008, 4:286-287.

65. Nunez M, Nunez E, Segur JM, Macule F, Quinto L, Hernandez MV, Vilalta C: The effect of an educational program to improve health-related quality of life in patients with osteoarthritis on waiting list for total knee replacement: A randomized study. ***Osteoarthritis Cart*** 2006, 14:279-285.

66. Rooks DS, Huang J, Bierbaum BE, Bolus SA, Rubano J, Connolly CE, Alpert S, Iversen MD, Katz JN: Effect of preoperative exercise on measures of functional status in men and women undergoing total hip and knee arthroplasty. ***Arthr Rheum*** 2006, 55:700-708.

67. Soni A, Mudge N, Joshi A, Wyatt M, Williamson L: Severe knee osteoarthritis: A study of combined acupuncture and physiotherapy vs home exercise advice in patients awaiting total knee arthroplasty. ***Rheumatology*** 2010, 49:i79-i80.

68. Taverner MG, Ward TL, Loughnan TE: Transcutaneous pulsed radiofrequency treatment in patients with painful knee awaiting total knee joint replacement. ***Clin J Pain*** 2010, 26:429-432.

69. Topp R, Swank AM, Quesada PM, Nyland J, Malkani A: The Effect of Prehabilitation Exercise on Strength and Functioning After Total Knee Arthroplasty. ***PM and R*** 2009, 1:729-735.

70. Weidenhielm L, Mattsson E, Broström LA, Wersäll-Robertsson E: Effect of preoperative physiotherapy in unicompartmental prosthetic knee replacement. ***Scand J Rehab Med*** 1993, 25:33-39.

71. Williamson L, Wyatt MR, Yein K, Melton JTK: Severe knee osteoarthritis: a randomized controlled trial of acupuncture, physiotherapy (supervised exercise) and standard management for patients awaiting knee replacement. ***Rheumatology*** 2007, 46:1445-1449.

72. Lin C-WC, March L, Crosbie J, Crawford R, Graves S, Naylor J, Harmer A, Jan S, Bennell K, Harris I *et al*: Maximum recovery after knee replacement--the MARKER study rationale and protocol. ***BMC Musculoskelet Disord*** 2009, 10:69.

73. Lastayo PC, Meier W, Marcus RL, Mizner R, Dibble L, Peters C: Reversing muscle and mobility deficits 1 to 4 years after tka: A pilot study. ***Clin Orthop Relat Res*** 2009, 467:1493-1500.

74. Trudelle-Jackson E, Smith SS: Effects of a late-phase exercise program after total hip arthroplasty: a randomized controlled trial. ***Arch Phys Med Rehabil*** 2004, 85:1056-1062.

75. Valtonen A, Poyhonen T, Sipila S, Heinonen A: Effects of aquatic resistance training on mobility limitation and lower-limb impairments after knee replacement. ***Arch Phys Med Rehabil*** 2010, 91:833-839.
